# 

**Published:** 1802-05

**Authors:** 


### To CORRESPONDENTS.

*We are not inattentive to the friendly hints we have received respecting personalities in Controversy. Some of our readers are so unreasonable as to desire the Journal to be made subservient to their particular pursuits; they cannot be* surprised *at our refusing to comply with such requests. We have no doubt that the communication on Cynanche Trachealis, in this Number, will impress all Practitioners with the importance of a periodical Publication which lays before the Professon,* monthly, *every new Discovery, and gives the* earliest notice of every EPIDEMIC.

